# Better understanding and prediction of antiviral peptides through primary and secondary structure feature importance

**DOI:** 10.1038/s41598-020-76161-8

**Published:** 2020-11-06

**Authors:** Abu Sayed Chowdhury, Sarah M. Reehl, Kylene Kehn-Hall, Barney Bishop, Bobbie-Jo M. Webb-Robertson

**Affiliations:** 1 Biological Sciences Division, Pacific Northwest National Laboratory, J4-18, P.O. Box 999, Richland, WA 99354 USA; 2 Computing and Analytics Division, Pacific Northwest National Laboratory, P.O. Box 999, Richland, WA 99354 USA; 3 School of Systems Biology, George Mason University, Manassas, VA 20110 USA; 4 National Center for Biodefense and Infectious Diseases, George Mason University, Manassas, VA 20110 USA; 5 Department of Biomedical Sciences and Pathobiology, Virginia Tech, Blacksburg, VA 24061 USA; 6 Department of Chemistry and Biochemistry, George Mason University, Manassas, VA 20110 USA

**Keywords:** Computational models, Machine learning, Software, Infection

## Abstract

The emergence of viral epidemics throughout the world is of concern due to the scarcity of available effective antiviral therapeutics. The discovery of new antiviral therapies is imperative to address this challenge, and antiviral peptides (AVPs) represent a valuable resource for the development of novel therapies to combat viral infection. We present a new machine learning model to distinguish AVPs from non-AVPs using the most informative features derived from the physicochemical and structural properties of their amino acid sequences. To focus on those features that are most likely to contribute to antiviral performance, we filter potential features based on their importance for classification. These feature selection analyses suggest that secondary structure is the most important peptide sequence feature for predicting AVPs. Our Feature-Informed Reduced Machine Learning for Antiviral Peptide Prediction (FIRM-AVP) approach achieves a higher accuracy than either the model with all features or current state-of-the-art single classifiers. Understanding the features that are associated with AVP activity is a core need to identify and design new AVPs in novel systems. The FIRM-AVP code and standalone software package are available at https://github.com/pmartR/FIRM-AVP with an accompanying web application at https://msc-viz.emsl.pnnl.gov/AVPR.

## Introduction

Zoonotic viruses such as Ebola virus, Zika virus, West Nile virus and recently severe acute respiratory syndrome coronavirus 2 (SARS-CoV-2) can cause life-threatening disease outbreaks due to their high genetic diversity, variety of routes for transmission, and ability to replicate efficiently and to persist in their hosts^1–4^. The control of viral disease continues to be a challenging task due to increased resistance to available antiviral therapies, which are limited, and the continual emergence of novel viral pathogens. Antiviral peptides (AVPs) are a subset of antimicrobial peptides and are a potential resource for the development of new potent therapeutics for preventing or treating viral infection. The ability of AVPs to target various aspects of the viral lifecycle, ranging from their attachment to host cells to their ability to impair viral replication within the cells has been the subject of multiple studies^5–13^. AVPs can be natural or synthetic, obtained by introducing chemical groups or non-natural amino acids into natural peptide sequences^4,13,14^. Considering AVPs in the design of new antiviral therapeutics is advantageous because it allows us to capitalize on their low molecular weight, low toxicity, high specificity and effectiveness, and minor side effects^15^. Machine learning is a powerful strategy for identifying AVPs by leveraging the ever-increasing data available in public databases, such as AVP Prediction (AVPpred)^16^, Antimicrobial Peptide Database (APD3)^17^, Collection of Antimicrobial Peptides (CAMPR3)^18^ and HIV inhibitory peptides database (HIPdb)^19^.

Researchers have previously developed machine learning models^16,20–25^ for predicting AVPs. Thakur et al.^16^ developed AVPpred, a web server for collecting and detecting highly effective AVPs. The authors used a support vector machine (SVM) to build two machine learning models based on amino acid composition (AAC) and physicochemical features. This was then extended to use a random forest (RF)-based model^20^, which was able to outperform the SVM utilized in AVPpred. The RF models were constructed using AAC, physicochemical properties, aggregation propensities of amino acids and secondary structure. Lissabet et al.^21^ developed a portable software version of the RF method called AntiVPP 1.0 that gives improved prediction accuracy. Qureshi et al.^22^ introduced a regression-based algorithm AVP-IC_50_Pred to predict AVP half maximal inhibitory concentration (IC_50_). Various peptide features such as AAC, binary profile, physicochemical properties, solvent accessibility were considered, and a number of machine learning techniques with individual and different combination of features were used to predict the IC_50_ value of the peptide sequences. Further, based on the assumption that AVPs have low sequence similarity the use of pseudo amino acid composition (PseAAC)^26^ was introduced as AVP peptide features in the AdaBoost machine learning model^23^. In recent years ensemble-based methods have been introduced, such as Meta-iAVP^25^ and PePred-Suite^24^. The Meta-iAVP approach uses machine learning to transform the feature space into a modified 6-dimensional predicted output vector, which then becomes the input data to the meta-classifier to predict the class of validation data set. PEPred-Suite is similar to Meta-iAVP where a RF is used as both the base and meta classifiers. Both Meta-iAVP and PEPred-Suite use these ensemble strategies to improve the AVP prediction accuracy.

The series of machine learning developments in AVP have to date focused on increasing the features that characterize a peptide and making minor modifications to the machine learning algorithm. They have not included feature reduction techniques that would determine the most relevant and non-redundant features from the initial set of input features. The performance of a machine learning model can rely heavily on using the most informative features, with the inclusion of non-informative features resulting in potential degradation in classifier performance. In the current study we identified the most important features by estimating Pearson's correlation coefficient and mean decrease of Gini index (MDGI) for all candidate features, which is a metric of feature importance based on the individual decision trees in a random forest model. The candidate features were generated from the physicochemical and secondary structure properties of a library of known AVP and non-AVP sequences. Subsequently, we applied a recursive feature elimination (RFE) algorithm in combination with the SVM to determine the order of importance of the different features. We evaluated multiple machine learning approaches, including SVM, RF and deep learning (DL) via multiple neural network architectures and hyperparameters, for training and testing purposes using our selected feature set. Our SVM-based method achieved the best test accuracy and Matthews correlation coefficient (MCC) values compared to the RF and DL approaches as well as outperformed AVPpred^16^ and Chang et al.’s method^20^. We packaged the resulting approach into a software tool called Feature-Informed Reduced Machine Learning for Antiviral Peptide Prediction (FIRM-AVP).

## Methods

### Training and testing data

We used the same experimentally validated dataset reported in AVPpred^16^ that has been used consistently since its introduction to evaluate AVP prediction models. It consists of a total of 1056 unique peptides. This set of peptides was distilled from a starting collection of 1245 peptides that were reduced to remove peptides with too high of similarity. Out of them, 604 sequences are highly effective (positive samples), and 452 sequences are minimally or non-effective AVPs (negative samples). These datasets were used for training and validating the machine learning model. To construct the training and independent test sets to benchmark our results with existing SVM and RF-based models we followed the same process as described previously^16,20^. This yields 544 and 407 positive and negative samples in the training dataset, respectively, and the validation/independent test set consisted of 60 and 45 positive and negative samples, respectively as defined by prior publications to assure accurate comparison. This validation set has similar overall viral diversity as the training set. On the AVPpred server there are additional peptides for the negative samples set, 544 in training set and 60 in the independent test set, however; these peptides have not been confirmed experimentally and thus are not included here.

### Feature generation

We combined several sets of features based on the peptide sequences: a 20D feature vector for AAC expressed as the percentage representation of a particular amino acid in a peptide; a 400D feature set was generated based on the dipeptide composition (DC) which represents the fraction of dipeptides within a peptide sequence; and the PseAAC and amphiphilic pseudo amino acid composition (APseAAC) proposed by Chou^26,27^ to incorporate sequence-order information. The dimension of the PseAAC feature vector is $20 + {\varvec{\lambda}} \times {\varvec{\omega}}$ where ${\varvec{\lambda}}$ is the discrete correlation factor and ${\varvec{\omega}}$ is the weight factor of the sequence information. In our case, we set ${\varvec{\lambda}}$ = 5 and ${\varvec{\omega}}$ = 0.05 by considering the minimum length of our collected AVP and non-AVP sequences. So, in the 25D PseAAC feature vector, the first 20 features are the traditional AAC and the other components are the rank-different correlation factors that represents the sequence-order information. We produced a $20 + 2{\varvec{\lambda}}$ i.e., 30D, APseAAC feature vector where the first 20 features are the basic AAC and the remaining components indicate the correlation factor for the physicochemical properties of peptides. We also utilized the composition, transition, and distribution (CTD) model^28–31^ to generate feature vectors for 8 physicochemical properties; hydrophobicity, normalized van der Waals volume, polarity, polarizability, charge, secondary structure, solvent accessibility and surface tension of peptide sequences. In the CTD model, amino acids are classified into three classes based on their physicochemical properties. For composition, we obtained 3D feature vector that give the fraction of each encoded class in a peptide sequence. A transition feature vector of 3D gives the transition of one class followed by another class and vice versa. We also obtained a 15D feature vector for distribution that indicates the percent distribution (i.e., 1%, 25%, 50%, 75% and 100%) of each class in a peptide sequence. As we have 8 physicochemical properties, the CTD model gives a (3 + 3 + 15)$\times$ 8 = 168D feature vector. Finally, we retrieved features from the secondary structure of peptide sequences. A total of six features were extracted from the location information, spatially consecutive states and segment sequences of the three main types of secondary structure; ${\varvec{\alpha}}$-helix, ${\varvec{\beta}}$-strand and ${\varvec{\gamma}}$-coil. The details of feature extraction from the CTD model and secondary structure information of amino acid sequences were explained in our previous works^32–34^. In summary, we generated 649 peptide sequence-based features listed in Table 1 using the R programming language (*ver* 4.0.0)^35^. We utilized the *protr* (*ver*. 1.6-2)^30^ and *DECIPHER* (*ver*. 2.14.0)^36^ packages to extract features from peptide sequences.

**Table 1:** List of 649 peptide features.

Peptide feature	Feature dimension
Amino acid composition	20
Dipeptide composition	400
Pseudo-amino acid composition	25
Amphiphilic pseudo-amino acid composition	30
Composition/transition/distribution	168
Secondary structure sequence	6

The DC feature vector (dipep_1, dipep_2, …, dipep_400) are the dipeptide composition (Supplementary Table S1) of the amino acids in order A, R, N, D, C, E, Q, G, H, I, L, K, M, F, P, S, T, W, Y, V. The PseAAC and APseAAC are the feature vectors (pseudo_1, pseudo_2, …, pseudo_25) and (amphipseudo_1, amphipseudo_2, …, amphipseudo_30), respectively. The composition feature vector (comp_1, comp_2, …, comp_24) and transition feature vector (tran_1, tran_2, …, tran_24) are the composition and transition values in the order-physicochemical property 1 (group 1), physicochemical property 1 (group 2), physicochemical property 1 (group 3) and so on. In the distribution feature vector (dist_1, dist_2, …, dist_120), the first 15D features are the group1, group2 and group3 distribution values for the first physiochemical property and so on. The physicochemical properties and their groups are listed as supplementary Table S2. Finally, in the 6D secondary structure feature vector, ss_1, ss_2 and ss_3 are the location-oriented features for the ${\varvec{\alpha}}$-helix, ${\varvec{\beta}}$-strand and ${\varvec{\gamma}}$-coil, respectively. The other three features ss_4, ss_5 and ss_6 gives the normalized maximum spatial consecutive ${\varvec{\alpha}}$-helix and ${\varvec{\beta}}$-strand in the secondary structure sequence, and occurrences of segmented sequences “${\varvec{\beta}}$-strand ${\varvec{\alpha}}$-helix ${\varvec{\beta}}$-strand” after ignoring ${\varvec{\gamma}}$-coil states from the secondary structure.

### Machine learning models

We utilized three machine learning approaches to train the AVP prediction model, traditional SVM and RF methods, as well as DL via multiple architectures and hyperparameters using the machine learning library, *caret* (*ver.* 6.0-86)^37^. For the DL, variations on the Multi-layer Perceptron were the most successful. These binary classification models were then used to classify the test set of peptides. Note that we tuned the SVM and RF models with the training dataset and used the best models for prediction. The SVM model was tuned using the radial basis function kernel with cost values of 4, 8, 16, 32, 64, and 128. The RF model was tuned with *ntree* values of 50, 100, 200, 300, 400 and 500 and *mtry* values of 2, 4, 8, 16, and 32. The final SVM model used a *cost* value of 8, and RF model was with *ntree* = 100 and *mtry* = 32, which was chosen as best models for the selected feature on the training data. We utilized the *e1071* (*ver.* 1.7-3)^38^ package to tune the models.

### Feature selection

The 649 features may contain redundant and information irrelavent to the classification of AVPs. To reduce the dimensions of the features we calculated the Pearson's correlation coefficient [using Eq. (1)] between two feature vectors $x$ and $y$ across all of the peptides to observe the linear correlation between features. Here $E$, $\mu$ and $\sigma$ are the expectation, mean and standard deviation values, respectively.

1 $$ \rho = \frac{{E\left[ {\left( {x - \mu_{x} } \right)\left( {y - \mu_{y} } \right)} \right]}}{{\sigma_{x} \sigma_{y} }}. $$

If the absolute value of the correlation between two features is greater than a threshold value, one of the two features were removed randomly from further consideration. We considered a range of correlation threshold from 0.7 to 0.95 in increments of 0.05. A correlation threshold was selected to optimize the Area Under a Receiver Operating Characteristic Curve (AUC) associated with the feature selection, which set the parameter to 0.85 and reduced the dataset to 568 features. We utilized the R *stats* package (*ver*. 3.6.2) to compute the Pearson correlation values between features.

As a next step, we computed mean decrease of Gini index (MDGI) using an RF model for the remaining features. We can find the feature importance using MDGI to measure the contribution of each feature to the homogeneity of the nodes and leaves in the RF model^39^. A node is considered as more pure in the RF model if the Gini index is closer to 0. The Gini index is calculated using Eq. (2) where we subtract the sum of the squared probabilities of each of the two classes from 1.

2 $$ Gini = 1 - \mathop \sum \nolimits_{i = 1}^{2} P_{i}^{2} . $$

So, the Gini index values of 0 and 1 indicate completely homogeneous data and completely heterogeneous data, respectively. To find the feature importance, whenever a feature is used to divide data at a node, we calculated the Gini index at the root node and at both the leaves. The difference in the Gini index of splitting root node and weighted Gini index of the child nodes was estimated to find the fall of Gini index values in a decision tree of the RF model^20^. For each feature, MDGI is the average value of all the decrease of Gini index over all the decision trees created in the RF model and higher MDGI value indicate elevated feature importance. Based on the MDGI we down-selected to 169 features with positive MDGI. The *randomForest* (*ver*. 4.6-14)^40^ package was used to estimate the MDGI values of the features.

### Recursive feature elimination

Following reduction of the number of features based on Pearson's correlation coefficient and MDGI values, we applied the RFE technique^41^ to the machine learning models using the training data for the reduced feature set to order the features by importance. RFE evaluates the training performance of a machine learning model for a feature set and gives the ranking of the features. We considered 10-fold cross validation with 5 repeats to evaluate the training performance of the machine learning models. We utilized *caret* (*ver*. 6.0-86)^37^ to implement the RFE algorithm.

### Performance measurement

We utilize the area under the receiver operating characteristic (ROC) curve (AUC) values to measure the training performance of the models via RFE for the reduced feature set. ROC curves use a combination of the true positive rate and false positive rate to provide a summary of the prediction capability of a machine learning model where a perfect classifier has an AUC of 1.0 and a random binary classifier will have an AUC of 0.5. We report the final test performance of our classifiers using the same metrics as previously reported for other AVP prediction algorithms, which include sensitivity, specificity, accuracy and MCC values [Eqs. (3–6)], where TP, TN, FP, and FN are true positives (positives accurately classified), true negatives (negatives accurately classified), false positives (negatives classified as positives), and false negatives (positives classified as negatives), respectively. The MCC value is used to evaluate the efficacy of a classifier as the number of positive and negative examples in the datasets is imbalanced and the range of this value is [− 1, 1]. Higher MCC value indicates better prediction.

3 $$ Sensitivity = \frac{TP}{{TP + FN}}, $$

4 $$ Specificity = \frac{TN}{{TN + FP}}, $$

5 $$ Accuracy = \frac{TP + TN}{{TP + TN + FP + FN}}, $$

6 $$ MCC = \frac{TP \times TN - FP \times FN}{{\sqrt {\left( {TP + FP} \right)\left( {TP + FN} \right)\left( {TN + FP} \right)\left( {TN + FN} \right)} }}. $$

### Data availability

All experimental data are available at https://github.com/pmartR/FIRM-AVP.

## Results

### AVP prediction performance

The performance of the FIRM-AVP SVM, RF and DL models were compared based on the standard metrics of sensitivity, specificity, accuracy, and MCC for their performance on the validation/independent dataset where a positive AVP peptide is defined as a probability of greater than 0.5 and a negative AVP as less than or equal to 0.5. Evaluating overall accuracy, we observe that the SVM and RF models have very high AUC values, 0.962 and 0.958, respectively. Table 2 details the results of the models for the 169 features based on our feature reduction. The SVM model achieved 92.4% accuracy and 0.84 MCC, which is better than the RF model. Both the SVM and RF machine learning approaches yield a posterior probability that represent the probability that a peptide is AVP given the data represented as the 169 features, or likewise the probability that a peptide is non-AVP. We evaluated the probabilities of the 60 positive AVPs for the SVM versus the RF and found that on average the strength of the prediction based on the probability for the SVM was larger than the RF by ~ 0.02 (paired t-test *p*-value ~ 0.14). Thus, there is marginal evidence that the SVM yields a more confident identification, but it is not statistically significant based on this data at a p-value threshold of 0.05. However, when evaluating the negative class there is a significant improvement gained by the SVM. The average non-AVP peptide is generally correctly classified with a larger probability by ~ 0.09 (*p*-value ~ 5E−5). This difference in strength of classification of the non-AVP class is what is largely driving the reduction in false positives for the SVM, which is observed in the specificity values reported in Table 2. The DL approaches were likely sub-optimal because while multiple nonlinearities exist in these data, the training examples are too few to both describe the nonlinearities and adequately generalize to new data. Evidence of such a conclusion is apparent in discrepancies between training and testing loss, even in the presence of regularization. Future work of importance is to grow and create more variety in the AVP benchmark dataset, which has not been updated in 8 years, which would aid in the application of more recent machine learning approaches.

**Table 2:** Performance comparison of our models with existing models on independent validation data.

Model	Sensitivity (%)	Specificity (%)	Accuracy (%)	MCC
FIRM-AVP (SVM)	93.3	91.1	92.4	0.84
FIRM-AVP (RF)	95.0	82.2	89.5	0.79
FIRM-AVP (DL)	91.7	80.0	86.7	0.73
AVP-649D (SVM)	95.0	82.2	89.5	0.79
AVP-649D (RF)	90.0	82.2	86.7	0.73
AVPcompo	83.3	88.9	85.7	0.72
AVPphysico	88.3	82.2	85.7	0.71
RFcompo + structure + agg	91.7	86.7	89.5	0.79
Meta-iAVP	95.2	96.7	93.2	0.90

For the independent test set, we then compared the performance of our FIRM-AVP SVM model with no feature reduction (AVP-649D), as well as with the AVPpred^16^ and the Chang et al.’s RF approaches (RFcompo + structure + agg)^20^ (Table 2). There is a clear increase in accuracy based on the reduced feature set from the full 649 features, for example our best performing SVM model increased the MCC from 0.79 to 0.84 by reducing to the highest importance features. In terms of prior analyses, the AVPcompo and AVPphysico are the models of AVPpred based on AAC and physicochemical features, respectively whereas RFcompo + structure + agg is the Chang et al.’s RF method that uses both (AAC), secondary structure and aggregation features. Chang et al.’s RF method outperforms AVPpred with an accuracy of 89.5% and 0.79 MCC value. However, our FIRM-AVP SVM models that is built on an optimized feature set performed better than either of these two methods in terms of accuracy and MCC and the FIRM-AVP RF model was similar to that of prior models. The most accurate model is Meta-iAVP^25^, which is based on an ensemble of machine learning algorithms. This however comes with a challenge in interpretation and gaining insight into the features that are driving antiviral activity as was the goal with FIRM-AVP. The same validation set run on each of the 6 machine learning algorithms separately have MCC values that range from 0.34 to 0.73, well below the FIRM-AVP using a single classifier on the optimized feature set.

### Recursive feature rankings

We performed RFE operations on the SVM model with the training data using 169 features from the initial feature selection with repetition measure the training performance of the SVM (in terms of AUC) via RFE algorithm. Note that the AUC values gradually decreased as features were removed from the model as depicted in Supplementary Fig. S1, and we obtained the highest AUC values of 0.89 and 0.92 for the SVM and RF models, respectively, by including all 169 features. This indicates that we do not need further feature reduction, and thus we utilize the RFE results to sort the importance of the features. Table 3 lists the top-5 features found after RFE analysis. Both secondary structure, composition and PseAAC features are in the top-5 features for both machine learning models. Peptide secondary structure features are identified as top ranked features in SVM and RF methods, respectively. All rankings of the selected features for both SVM and RF models are listed in Supplementary Table S3.

**Table 3:** Top-5 features obtained in SVM and RF methods from RFE analysis.

Feature rank	Features for SVM	Features for RF
1	**Location oriented feature for **$\alpha$**-helix**	Distribution (25% residues) feature for positive charge (group 1)
2	**PseAAC feature for leucine (L) amino acid**	Composition feature for intermediate solvent accessibility (group 3)
3	PseAAC feature for *isoleucine* (I) amino acid	**PseAAC feature for leucine (L) amino acid**
4	**PseAAC feature for lysine (K) amino acid**	**Location oriented feature for **$\alpha$**-helix**
5	Composition feature for neutral hydrophobicity (group 2)	**PseAAC feature for lysine (K) amino acid**

Common features are highlighted in bold.

### Software tool and user's manual

**Figure 1:**
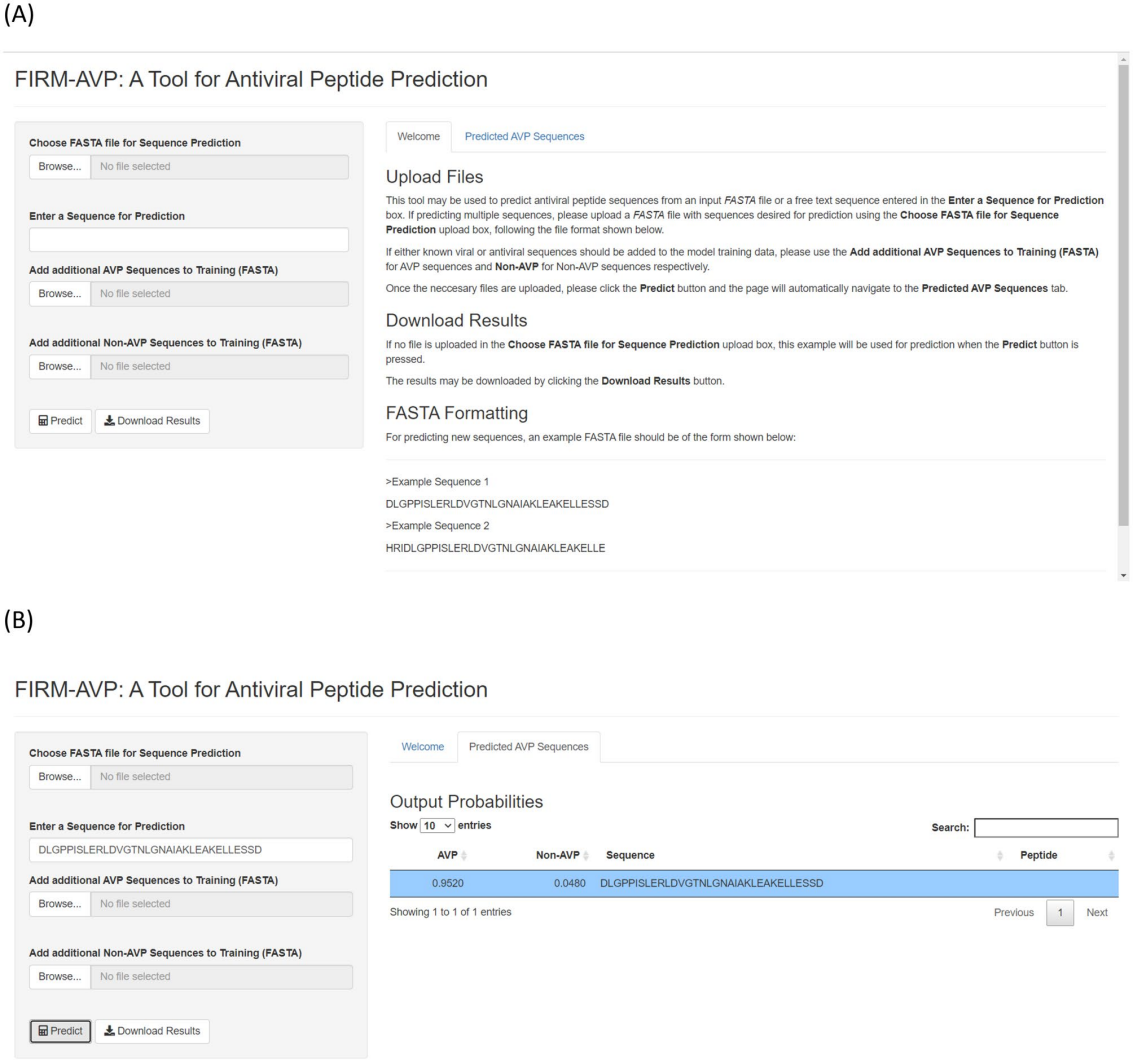
Online FIRM-AVP software interface (https://msc-viz.emsl.gov/AVPR/). Where (**A**) is the starting page that allows users to either paste in a single peptide sequence or upload a FASTA file containing a collection of peptide sequences. Example sequences and files are given. (**B**) The probability of AVP versus non-AVP is returned for each sequence based on the pasted peptide sequence or the uploaded FASTA file.

We developed the standalone software tool, FIRM-AVP based on the SVM algorithm. The open source software are available at https://github.com/pmartR/FIRM-AVP. Additionally, a web-based version of the software is available at https://msc-viz.emsl.gov/AVPR/ . To use the web application the users need to provide either a single peptide sequence or a FASTA file of peptide sequences to be analyzed and predictions will be returned that include the probability that a peptide sequence is antiviral (Fig. 1). As previously mentioned, a current limitation in AVP prediction is the scale of the data available on which to build predictive models. To make the software more useful for those working on improving the algorithm via collecting additional training data, the software provides the user an option to add new known AVP and non-AVP sequences to retrain the machine learning model. A simple page refresh will reset the model. The graphical user interface and options the web application provides are shown in Fig. 1. The feature generation and selection components of the software were implemented using R. The graphical user interface design and implementation were created using the R web application framework *shiny* (*ver*. 1.4.0.2)^42^.

## Discussion

Identifying potential AVPs is of great importance for the discovery of new drugs to treat viral infection. In this work, we introduced a machine learning model for predicting AVPs using a core set of 169 features identified via correlation and machine learning analyses. Our SVM and RF models were developed based on the features generated from the AAC, DC, PseAAC, APseAAC, CTD, and predicted secondary structure properties of peptide sequences. To verify the effectiveness of our best feature sets, we tested the performance of our models using an independent dataset that included the same validation/independent as prior methods^16,20^. We achieved higher accuracies and MCC values relative to single classifier models that did not include feature reduction, as well as existing published models, demonstrating the effectiveness of the feature selection approach. The software tool FIRM-AVP based on our approach is publicly available for user with flexible options to not only make predictions, but to update the underlying prediction model. The need for more training data was a limiting factor to the DL approach, which had lower overall accuracy than the SVM and RF approaches.

We evaluated multivariate feature importance using our selected feature sets via RFE. Secondary structure and distribution features were identified as top ranked features in our SVM and RF models, respectively. Location oriented features for $\alpha$-helix conformation and distributional features associated with positive charge as the most important features of the machine learning models. The PSeAAC feature for leucine and lysine amino acids were also important in distinguishing AVP and non-AVP sequences. The location oriented feature for $\alpha$-helix and PSeAAC features for leucine and lysine amino acids support the abundance of the $\alpha$-helix structure, and leucine and lysine residues in AVPs that were claimed in the RF-based method^20^ and HIPdb^19^. The observed significance of α-helical structure is consistent with the fact that many known antimicrobial peptides exhibit varied degrees of helical conformation and spatial partitioning of cationic and hydrophobic residues^43^. Here, both the SVM and RF approaches establish helix distributional features that are associated with antiviral peptides^44,45^. How these properties factor in peptide antiviral activity is not clear, however they are known to contribute to their interactions with cell membranes.

The discovery of new antiviral therapies is imperative to address the challenge of new viral epidemics and AVPs can be a valuable resource for the development of novel therapies to combat viral infection. One of the core needs is not only improving the accuracy of AVP prediction models, but also building explainable models that can aid in understanding the fundamental multivariate properties that are associated with anti-viral activity. This is a necessary step in the design of AVP design for novel viral systems.

## Supplementary information

Supplementary Information.
