# Supplementary material for: Better understanding and prediction of antiviral peptides through primary and secondary structure feature importance
Source: Sci Rep. 2020 Nov 6;10:19260. doi: 10.1038/s41598-020-76161-8 (PMC7648056; doi:10.1038/s41598-020-76161-8)
Supplement: Supplementary file 1 — Supplementary Information. [file 41598_2020_76161_MOESM1_ESM.docx]

**Better Understanding and Prediction of Antiviral Peptides through Primary and Secondary Structure Feature Importance**

Abu Sayed Chowdhury^1^, Sarah M. Reehl^2^, Kylene Kehn-Hall^3,4,5^, Barney Bishop^6^, Bobbie-Jo M. Webb-Robertson^1,*^

^1^Biological Sciences Division, Pacific Northwest National Laboratory, P.O. Box 999, Richland, WA 99354, USA,

^2^Computing & Analytics Division, Pacific Northwest National Laboratory, P.O. Box 999, Richland, WA 99354, USA,

^3^School of Systems Biology, George Mason University, Manassas, VA 20110, USA,

^4^National Center for Biodefense and Infectious Diseases, George Mason University, Manassas, VA 20110, USA

^5^Department of Biomedical Sciences and Pathobiology, Virginia Tech, Blacksburg, VA 24061, USA

^6^Department of Chemistry and Biochemistry, George Mason University, Manassas, VA 20110, USA

^*^Corresponding author: bj@pnnl.gov

| 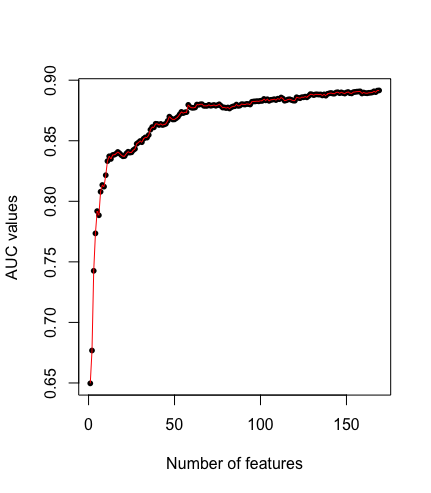   1. AUC values for the subsets of 169 features |
| --- |

**Figure S1.** Illustrating the performance of SVM for the training dataset via RFE.

**Table S1.** List of 400 dipeptides

| **No** | **Dipeptide** |
| --- | --- |
| 1 | AA |
| 2 | RA |
| 3 | NA |
| 4 | DA |
| 5 | CA |
| 6 | EA |
| 7 | QA |
| 8 | GA |
| 9 | HA |
| 10 | IA |
| 11 | LA |
| 12 | KA |
| 13 | MA |
| 14 | FA |
| 15 | PA |
| 16 | SA |
| 17 | TA |
| 18 | WA |
| 19 | YA |
| 20 | VA |
| 21 | AR |
| 22 | RR |
| 23 | NR |
| 24 | DR |
| 25 | CR |
| 26 | ER |
| 27 | QR |
| 28 | GR |
| 29 | HR |
| 30 | IR |
| 31 | LR |
| 32 | KR |
| 33 | MR |
| 34 | FR |
| 35 | PR |
| 36 | SR |
| 37 | TR |
| 38 | WR |
| 39 | YR |
| 40 | VR |
| 41 | AN |
| 42 | RN |
| 43 | NN |
| 44 | DN |
| 45 | CN |
| 46 | EN |
| 47 | QN |
| 48 | GN |
| 49 | HN |
| 50 | IN |
| 51 | LN |
| 52 | KN |
| 53 | MN |
| 54 | FN |
| 55 | PN |
| 56 | SN |
| 57 | TN |
| 58 | WN |
| 59 | YN |
| 60 | VN |
| 61 | AD |
| 62 | RD |
| 63 | ND |
| 64 | DD |
| 65 | CD |
| 66 | ED |
| 67 | QD |
| 68 | GD |
| 69 | HD |
| 70 | ID |
| 71 | LD |
| 72 | KD |
| 73 | MD |
| 74 | FD |
| 75 | PD |
| 76 | SD |
| 77 | TD |
| 78 | WD |
| 79 | YD |
| 80 | VD |
| 81 | AC |
| 82 | RC |
| 83 | NC |
| 84 | DC |
| 85 | CC |
| 86 | EC |
| 87 | QC |
| 88 | GC |
| 89 | HC |
| 90 | IC |
| 91 | LC |
| 92 | KC |
| 93 | MC |
| 94 | FC |
| 95 | PC |
| 96 | SC |
| 97 | TC |
| 98 | WC |
| 99 | YC |
| 100 | VC |
| 101 | AE |
| 102 | RE |
| 103 | NE |
| 104 | DE |
| 105 | CE |
| 106 | EE |
| 107 | QE |
| 108 | GE |
| 109 | HE |
| 110 | IE |
| 111 | LE |
| 112 | KE |
| 113 | ME |
| 114 | FE |
| 115 | PE |
| 116 | SE |
| 117 | TE |
| 118 | WE |
| 119 | YE |
| 120 | VE |
| 121 | AQ |
| 122 | RQ |
| 123 | NQ |
| 124 | DQ |
| 125 | CQ |
| 126 | EQ |
| 127 | QQ |
| 128 | GQ |
| 129 | HQ |
| 130 | IQ |
| 131 | LQ |
| 132 | KQ |
| 133 | MQ |
| 134 | FQ |
| 135 | PQ |
| 136 | SQ |
| 137 | TQ |
| 138 | WQ |
| 139 | YQ |
| 140 | VQ |
| 141 | AG |
| 142 | RG |
| 143 | NG |
| 144 | DG |
| 145 | CG |
| 146 | EG |
| 147 | QG |
| 148 | GG |
| 149 | HG |
| 150 | IG |
| 151 | LG |
| 152 | KG |
| 153 | MG |
| 154 | FG |
| 155 | PG |
| 156 | SG |
| 157 | TG |
| 158 | WG |
| 159 | YG |
| 160 | VG |
| 161 | AH |
| 162 | RH |
| 163 | NH |
| 164 | DH |
| 165 | CH |
| 166 | EH |
| 167 | QH |
| 168 | GH |
| 169 | HH |
| 170 | IH |
| 171 | LH |
| 172 | KH |
| 173 | MH |
| 174 | FH |
| 175 | PH |
| 176 | SH |
| 177 | TH |
| 178 | WH |
| 179 | YH |
| 180 | VH |
| 181 | AI |
| 182 | RI |
| 183 | NI |
| 184 | DI |
| 185 | CI |
| 186 | EI |
| 187 | QI |
| 188 | GI |
| 189 | HI |
| 190 | II |
| 191 | LI |
| 192 | KI |
| 193 | MI |
| 194 | FI |
| 195 | PI |
| 196 | SI |
| 197 | TI |
| 198 | WI |
| 199 | YI |
| 200 | VI |
| 201 | AL |
| 202 | RL |
| 203 | NL |
| 204 | DL |
| 205 | CL |
| 206 | EL |
| 207 | QL |
| 208 | GL |
| 209 | HL |
| 210 | IL |
| 211 | LL |
| 212 | KL |
| 213 | ML |
| 214 | FL |
| 215 | PL |
| 216 | SL |
| 217 | TL |
| 218 | WL |
| 219 | YL |
| 220 | VL |
| 221 | AK |
| 222 | RK |
| 223 | NK |
| 224 | DK |
| 225 | CK |
| 226 | EK |
| 227 | QK |
| 228 | GK |
| 229 | HK |
| 230 | IK |
| 231 | LK |
| 232 | KK |
| 233 | MK |
| 234 | FK |
| 235 | PK |
| 236 | SK |
| 237 | TK |
| 238 | WK |
| 239 | YK |
| 240 | VK |
| 241 | AM |
| 242 | RM |
| 243 | NM |
| 244 | DM |
| 245 | CM |
| 246 | EM |
| 247 | QM |
| 248 | GM |
| 249 | HM |
| 250 | IM |
| 251 | LM |
| 252 | KM |
| 253 | MM |
| 254 | FM |
| 255 | PM |
| 256 | SM |
| 257 | TM |
| 258 | WM |
| 259 | YM |
| 260 | VM |
| 261 | AF |
| 262 | RF |
| 263 | NF |
| 264 | DF |
| 265 | CF |
| 266 | EF |
| 267 | QF |
| 268 | GF |
| 269 | HF |
| 270 | IF |
| 271 | LF |
| 272 | KF |
| 273 | MF |
| 274 | FF |
| 275 | PF |
| 276 | SF |
| 277 | TF |
| 278 | WF |
| 279 | YF |
| 280 | VF |
| 281 | AP |
| 282 | RP |
| 283 | NP |
| 284 | DP |
| 285 | CP |
| 286 | EP |
| 287 | QP |
| 288 | GP |
| 289 | HP |
| 290 | IP |
| 291 | LP |
| 292 | KP |
| 293 | MP |
| 294 | FP |
| 295 | PP |
| 296 | SP |
| 297 | TP |
| 298 | WP |
| 299 | YP |
| 300 | VP |
| 301 | AS |
| 302 | RS |
| 303 | NS |
| 304 | DS |
| 305 | CS |
| 306 | ES |
| 307 | QS |
| 308 | GS |
| 309 | HS |
| 310 | IS |
| 311 | LS |
| 312 | KS |
| 313 | MS |
| 314 | FS |
| 315 | PS |
| 316 | SS |
| 317 | TS |
| 318 | WS |
| 319 | YS |
| 320 | VS |
| 321 | AT |
| 322 | RT |
| 323 | NT |
| 324 | DT |
| 325 | CT |
| 326 | ET |
| 327 | QT |
| 328 | GT |
| 329 | HT |
| 330 | IT |
| 331 | LT |
| 332 | KT |
| 333 | MT |
| 334 | FT |
| 335 | PT |
| 336 | ST |
| 337 | TT |
| 338 | WT |
| 339 | YT |
| 340 | VT |
| 341 | AW |
| 342 | RW |
| 343 | NW |
| 344 | DW |
| 345 | CW |
| 346 | EW |
| 347 | QW |
| 348 | GW |
| 349 | HW |
| 350 | IW |
| 351 | LW |
| 352 | KW |
| 353 | MW |
| 354 | FW |
| 355 | PW |
| 356 | SW |
| 357 | TW |
| 358 | WW |
| 359 | YW |
| 360 | VW |
| 361 | AY |
| 362 | RY |
| 363 | NY |
| 364 | DY |
| 365 | CY |
| 366 | EY |
| 367 | QY |
| 368 | GY |
| 369 | HY |
| 370 | IY |
| 371 | LY |
| 372 | KY |
| 373 | MY |
| 374 | FY |
| 375 | PY |
| 376 | SY |
| 377 | TY |
| 378 | WY |
| 379 | YY |
| 380 | VY |
| 381 | AV |
| 382 | RV |
| 383 | NV |
| 384 | DV |
| 385 | CV |
| 386 | EV |
| 387 | QV |
| 388 | GV |
| 389 | HV |
| 390 | IV |
| 391 | LV |
| 392 | KV |
| 393 | MV |
| 394 | FV |
| 395 | PV |
| 396 | SV |
| 397 | TV |
| 398 | WV |
| 399 | YV |
| 400 | VV |

**Table S2.** Composition/transition/distribution (CTD) model

| Hydrophobicity | Polar | Neutral | Hydrophobicity |
| --- | --- | --- | --- |
|  | R, K, E, D, Q, N | G, A, S, T, P, H, Y | C, L, V, I, M, F, W |
| Normalized van der Waals Volume | 0-2.78 | 2.95-4.0 | 4.03-8.08 |
|  | G, A, S, T, P, D, C | N, V, E, Q, I, L | M, H, K, F, R, Y, W |
| Polarity | 4.9-6.2 | 8.0-9.2 | 10.4-13.0 |
|  | L, I, F, W, C, M, V, Y | P, A, T, G, S | H, Q, R, K, N, E, D |
| Polarizability | 0-1.08 | 0.128-0.186 | 0.219-0.409 |
|  | G, A, S, D, T | C, P, N, V, E, Q, I, L | K, M, H, F, R, Y, W |
| Charge | Positive | Neutral | Negative |
|  | K, R | A, N, C, Q, G, H, I, L, M, F, P, S, T, W, Y, V | D, E |
| Secondary Structure | Helix | Strand | Coil |
|  | E, A, L, M, Q, K, R, H | V, I, Y, C, W, F, T | G, N, P, S, D |
| Solvent Accessibility | Buried | Exposed | Intermediate |
|  | A, L, F, C, G, I, V, W | R, K, Q, E, N, D | M, S, P, T, H, Y |

**Table S3.** Ranking of the features (SVM and RF models)

| **Rank** | **Feature (SVM model)** | **Feature (RF model)** |
| --- | --- | --- |
| 1 | ss_1 | dist_62 |
| 2 | pseudo_11 | comp_21 |
| 3 | pseudo_10 | pseudo_11 |
| 4 | pseudo_12 | ss_1 |
| 5 | comp_2 | pseudo_12 |
| 6 | dist_62 | pseudo_18 |
| 7 | pseudo_18 | pseudo_10 |
| 8 | comp_21 | comp_3 |
| 9 | comp_3 | aac_12 |
| 10 | aac_10 | comp_19 |
| 11 | dist_46 | amphipseudo_21 |
| 12 | amphipseudo_27 | aac_17 |
| 13 | comp_4 | amphipseudo_27 |
| 14 | aac_18 | dist_76 |
| 15 | dist_76 | comp_22 |
| 16 | dist_32 | dist_34 |
| 17 | dist_108 | amphipseudo_25 |
| 18 | amphipseudo_25 | comp_18 |
| 19 | dist_15 | tran_17 |
| 20 | dist_2 | dist_47 |
| 21 | aac_11 | dist_46 |
| 22 | dist_17 | amphipseudo_23 |
| 23 | aac_12 | pseudo_22 |
| 24 | dist_16 | dist_106 |
| 25 | dipep_211 | comp_2 |
| 26 | dist_91 | dist_91 |
| 27 | tran_1 | dist_8 |
| 28 | comp_18 | pseudo_23 |
| 29 | dist_73 | dist_108 |
| 30 | dist_8 | comp_10 |
| 31 | comp_10 | dist_16 |
| 32 | comp_19 | dist_1 |
| 33 | tran_21 | dist_18 |
| 34 | dist_1 | dist_94 |
| 35 | aac_15 | tran_23 |
| 36 | dist_18 | dist_77 |
| 37 | dist_3 | pseudo_25 |
| 38 | dist_30 | amphipseudo_24 |
| 39 | dist_77 | tran_6 |
| 40 | dist_47 | dist_32 |
| 41 | dist_120 | tran_12 |
| 42 | aac_17 | dist_2 |
| 43 | comp_5 | amphipseudo_29 |
| 44 | amphipseudo_21 | tran_24 |
| 45 | dist_9 | dist_3 |
| 46 | dist_107 | aac_10 |
| 47 | dist_106 | amphipseudo_22 |
| 48 | tran_17 | dist_17 |
| 49 | dist_78 | dist_9 |
| 50 | dist_103 | dist_63 |
| 51 | dipep_111 | dist_7 |
| 52 | comp_22 | aac_3 |
| 53 | dist_70 | pseudo_24 |
| 54 | dist_12 | comp_6 |
| 55 | dist_11 | dist_73 |
| 56 | dist_102 | comp_4 |
| 57 | comp_16 | pseudo_2 |
| 58 | tran_20 | tran_1 |
| 59 | dist_38 | amphipseudo_30 |
| 60 | dist_113 | dist_12 |
| 61 | dist_93 | dist_79 |
| 62 | dist_63 | amphipseudo_26 |
| 63 | pseudo_22 | dist_117 |
| 64 | dist_89 | dist_116 |
| 65 | pseudo_25 | dist_107 |
| 66 | dist_27 | dist_78 |
| 67 | dist_7 | dist_11 |
| 68 | tran_6 | dist_4 |
| 69 | dist_97 | tran_21 |
| 70 | amphipseudo_24 | dist_113 |
| 71 | dist_52 | dist_52 |
| 72 | amphipseudo_23 | pseudo_16 |
| 73 | dist_99 | aac_11 |
| 74 | dist_100 | dist_15 |
| 75 | dist_79 | comp_24 |
| 76 | comp_24 | dist_38 |
| 77 | dipep_220 | dist_50 |
| 78 | comp_11 | tran_22 |
| 79 | dist_28 | tran_16 |
| 80 | dist_14 | dist_22 |
| 81 | dist_67 | dist_97 |
| 82 | dist_117 | pseudo_5 |
| 83 | dist_41 | tran_20 |
| 84 | aac_8 | dist_83 |
| 85 | dist_24 | dist_114 |
| 86 | aac_20 | dist_100 |
| 87 | tran_22 | dist_30 |
| 88 | pseudo_16 | dist_70 |
| 89 | tran_2 | dist_112 |
| 90 | dist_85 | comp_11 |
| 91 | dist_22 | comp_16 |
| 92 | tran_16 | comp_23 |
| 93 | tran_5 | comp_17 |
| 94 | dist_13 | tran_13 |
| 95 | tran_14 | dist_67 |
| 96 | dist_116 | pseudo_21 |
| 97 | amphipseudo_22 | dipep_51 |
| 98 | aac_6 | aac_18 |
| 99 | pseudo_24 | dist_89 |
| 100 | dist_94 | dist_86 |
| 101 | dist_119 | aac_2 |
| 102 | dist_82 | comp_1 |
| 103 | tran_19 | dist_10 |
| 104 | dist_105 | dist_61 |
| 105 | dist_53 | dist_27 |
| 106 | pseudo_14 | dist_109 |
| 107 | amphipseudo_29 | dist_103 |
| 108 | aac_19 | dist_53 |
| 109 | aac_2 | tran_2 |
| 110 | pseudo_1 | dist_102 |
| 111 | dist_10 | tran_19 |
| 112 | dist_114 | comp_5 |
| 113 | pseudo_2 | pseudo_1 |
| 114 | dipep_32 | dist_65 |
| 115 | amphipseudo_26 | dist_93 |
| 116 | comp_14 | dist_87 |
| 117 | pseudo_4 | dist_85 |
| 118 | dist_29 | comp_14 |
| 119 | dist_50 | dist_99 |
| 120 | pseudo_3 | dist_115 |
| 121 | tran_23 | dist_55 |
| 122 | dist_34 | dipep_211 |
| 123 | dist_23 | dist_24 |
| 124 | dipep_340 | comp_15 |
| 125 | tran_18 | dist_120 |
| 126 | tran_12 | comp_13 |
| 127 | dist_26 | tran_3 |
| 128 | pseudo_5 | dist_56 |
| 129 | amphipseudo_30 | tran_4 |
| 130 | comp_15 | dist_82 |
| 131 | dist_55 | dist_84 |
| 132 | comp_23 | dist_26 |
| 133 | aac_3 | dist_41 |
| 134 | aac_1 | dist_23 |
| 135 | dist_56 | aac_8 |
| 136 | comp_1 | aac_20 |
| 137 | dist_109 | aac_16 |
| 138 | tran_13 | dist_119 |
| 139 | aac_7 | dist_71 |
| 140 | aac_9 | dist_68 |
| 141 | aac_4 | pseudo_20 |
| 142 | tran_24 | dist_118 |
| 143 | dist_84 | tran_5 |
| 144 | dist_88 | tran_14 |
| 145 | pseudo_21 | dist_90 |
| 146 | dist_65 | dist_13 |
| 147 | dist_4 | dist_72 |
| 148 | dist_87 | dist_25 |
| 149 | pseudo_23 | tran_18 |
| 150 | dist_115 | tran_11 |
| 151 | dist_86 | dist_105 |
| 152 | dist_90 | dipep_340 |
| 153 | dist_25 | aac_15 |
| 154 | dist_71 | pseudo_3 |
| 155 | dist_118 | pseudo_4 |
| 156 | tran_11 | dist_28 |
| 157 | comp_6 | dist_29 |
| 158 | aac_16 | aac_1 |
| 159 | dist_83 | pseudo_14 |
| 160 | dist_68 | aac_19 |
| 161 | tran_3 | aac_6 |
| 162 | tran_4 | aac_4 |
| 163 | comp_13 | dist_14 |
| 164 | dist_72 | dist_88 |
| 165 | dist_61 | aac_9 |
| 166 | comp_17 | aac_7 |
| 167 | pseudo_20 | dipep_220 |
| 168 | dist_112 | dipep_111 |
| 169 | dipep_51 | dipep_32 |
